# Phase Equilibria of Ternary and Quaternary Systems Containing Diethyl Carbonate with Water

**DOI:** 10.1007/s10953-014-0209-9

**Published:** 2014-08-22

**Authors:** Yao Chen, Caiyu Wen, Xiaoming Zhou, Jun Zeng

**Affiliations:** Department of Chemistry, Jinan University, Guangzhou, 510632 Guangdong People’s Republic of China

**Keywords:** Diethyl carbonate mixtures, Tie lines, Modified UNIQUAC model, Extended UNIQUAC model

## Abstract

In this study liquid phase equilibrium compositions were measured at 298.15 K under atmospheric pressure for (water + propan-1-ol + diethyl carbonate (DEC) + benzene or cyclohexane or heptane) quaternary systems and (water + DEC + propan-1-ol or benzene or cyclohexane) ternary systems. Good correlation of the experimental LLE data was seen for the measured systems by both modified and extended UNIQUAC models. The solubility of DEC in aqueous and organic phases is shown by equilibrium distribution coefficients calculated from the LLE data.

## Introduction

Some primary alcohols, ethers and alkyl carbonates are used for gasoline additives as octane boosters [1]. Diethyl carbonate (DEC) is one of the important benign chemicals. It is considered as being environment friendly. Compared with the tradition gasoline additive methyl *tert*-butyl ether (MTBE), DEC has a higher oxygen content, and it is both low in toxicity and biodegrades quickly. Adding DEC to gasoline can not only enhance the octane rating, but it also reduces the content of alkenes and aromatic hydrocarbons which can give rise to environmental pollution. In recent years these is considerable interest in using dimethyl carbonate or DEC to replace MTBE for meeting the oxygenate specifications on gasoline. The components of the gasoline are mainly C_4_–C_12_ alkanes, cyclanes, olefins and aromatic hydrocarbon. Considering the pollution from gasoline in the aqueous environment, such as the migration of gasoline additives from engines, the leakage of oil tanks in gasoline stations and in the process of transportation and storage, we considered the effect of adding water in these systems to simulate the real environment. Liquid–liquid equilibrium (LLE) data are necessary and important basic data, which play an important role in understanding phase behavior for the multicomponent systems. To provide accurate solubility data and check thermodynamic models, people have studied LLE and physical properties of mixtures containing alkyl carbonates [2–6].

Here, we report LLE for three ternary systems (water + DEC + propan-1-ol or benzene or cyclohexane) and three quaternary systems (water + propan-1-ol + DEC + benzene or cyclohexane or heptane) at *T* = 298.15 K and ambient pressure. The measured LLE results were correlated by using the modified and extended UNIQUAC models [7, 8] having binary, ternary, and quaternary parameters. The binary energy parameters for completely miscible binary mixtures were obtained from experimental vapor–liquid equilibria (VLE) data [9–14]. The binary energy parameters for partially miscible binary mixtures were obtained from mutual solubility data [15–17]. The ternary and quaternary parameters were obtained from the experimental LLE data. The binary, ternary and quaternary parameters are required to represent accurately the quaternary LLE data.

## Experimental

### Materials

The chemicals used in these experiments were DEC, benzene, propan-1-ol, cyclohexane, water and heptane. The mass fraction purity reported by the manufacturers was better than 0.990. Bi-distilled water was used in this work. The specifications of the chemicals used in this work are given in Table 1.

**Table 1:** Purities and suppliers of the chemicals

Chemical	Mass fraction purity	Supplier
Benzene (AR)^a^	0.9920	Guangzhou-Reagent Co. (Guangzhou, China)
Cyclohexane (AR)	0.9910	Tianjin-Reagent Co. (Tianjin, China)
DEC (AR)	0.9945	Aladdin-Reagent Co.(Shanghai, China)
Heptanes (AR)	0.9965	Fuyu-Reagent Co. (Tianjin, China)
Propan-1-ol (AR)	0.9950	Tianjin-Reagent Co. (Tianjin, China)

^a^AR means analytical reagent

### Procedures

Measurements were carried out on a binary test system (DMC + water) [6] at 298.15 K to validate the experimental technique by determining the mole fraction of DMC in water solution and comparing it with literature value. The estimated error in mole fraction is less than ±5 × 10^−4^.

**Fig. 1:**
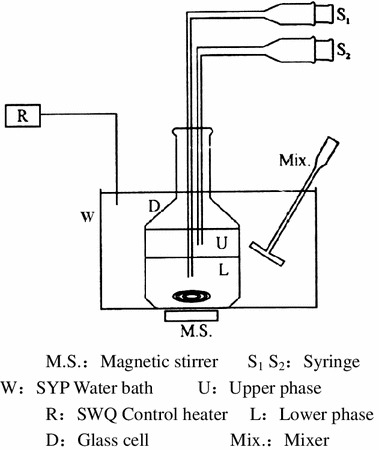
Schematic diagram for LLE measurement

LLE **e**xperiments were carried out in a glass cell. The experimental apparatus is schematically shown in Fig. 1. The equilibrium cell was a 120 cm^3^ glass cell. The temperature of the cell liquids was measured by a thermocouple. For maintaining temperature, about 70 cm^3^ of each mixture was loaded into the equilibrium glass cell placed in a thermostatted water bath. The temperature of the water bath was controlled at *T* = 298.15 K, and the temperature uncertainty was ± 0.05 K. Contents were stirred well by a magnetic stirrer for about 3 h and were then allowed to settle for more than 8 h at constant temperature, which was long enough to reach thermodynamic equilibrium. For analysis, the samples of upper and lower phases were withdrawn with the help of syringes. Compositional analysis of samples was carried out in a gas chromatograph (Shimadzu Analyses Apparatus Co., Shuzhou, China, GC-14C) with a thermal conductivity detector. The analysis was performed with a Porapak QS packed column (3 mm × 2.5 m). The detector and injector temperatures were kept at 510.15 and 490.15 K, respectively. Hydrogen was used as carrier gas at a rate of 60 mL·min^−1^ throughout the column, and the column inlet pressure was 0.1 MPa. A chromatopac (N2000) was used to detect the peak areas of the components. Each sample was analyzed at least three times and average values obtained. The accuracy in these experimental measurements was found to be (±6 × 10^−4^) for the mole fraction.

## Calculation Procedure and Results Discussion

**Fig. 2:**
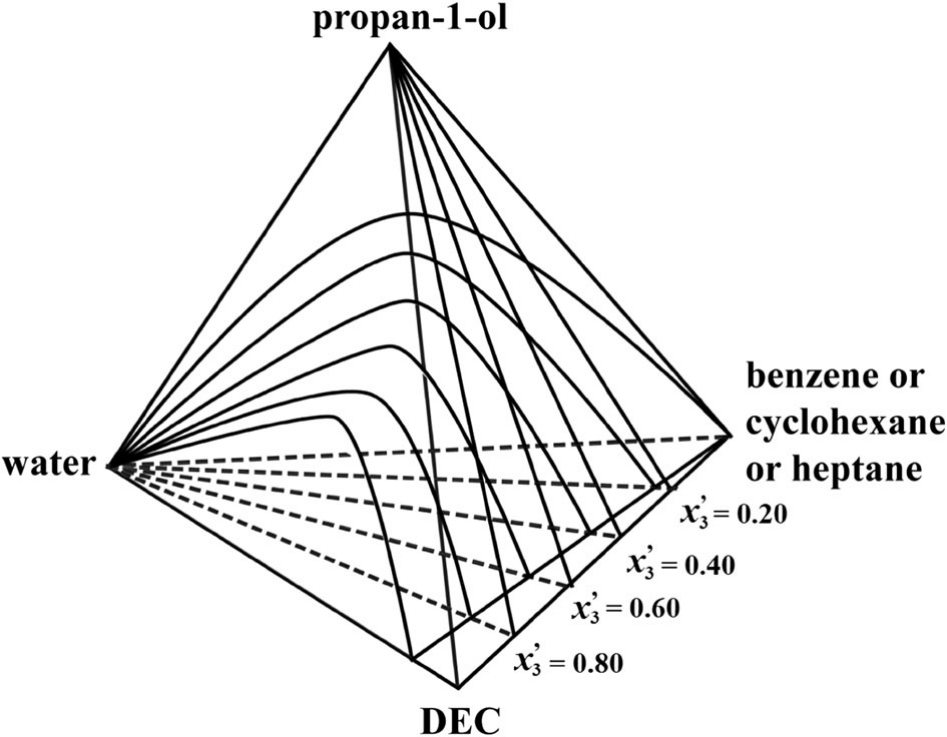
Phase equilibria of (water + propan-1-ol + DEC + benzene or cyclohexane or heptane); *x*_3_^′^ denotes a quaternary section plane

The experimental LLE results for the three ternary systems (water + propan-1-ol + DEC), (water + DEC + benzene) and (water + DEC + cyclohexane) measured at *T* = 298.15 K are reported in Tables 2, 3 and 4. Tables 5, 6 and 7 list the experimental LLE results for the three quaternary systems (water + propan-1-ol + DEC + benzene), (water + propan-1-ol + DEC + cyclohexane) and (water + propan-1-ol + DEC + heptane) at *T* = 298.15 K. Figure 2 shows a tetrahedron to depict three planes of the quaternary LLE for the (water + propan-1-ol + DEC + benzene), (water + propan-1-ol + DEC + cyclohexane) and (water + propan-1-ol + DEC + heptane) systems. Each quaternary system consists of three ternary systems. For example, the quaternary system (water + propan-1-ol + DEC + benzene) is comprised of three ternary subsystems (water + propan-1-ol + DEC), (water + propan-1-ol + benzene) and (water + DEC + benzene).

**Table 2:** Experimental (liquid + liquid) equilibrium data for the ternary system of water (1) + propan-1-ol (2) + diethyl carbonate (3) for mole fractions *x* at the temperature *T* = 298.15 K

Organic phase			Aqueous phase		
*x*_1_	*x*_2_	*x*_3_	*x*_1_	*x*_2_	*x*_3_
0.0475	0.0000	0.9525	0.9978	0.0000	0.0022
0.0178	0.1073	0.8749	0.9767	0.0176	0.0057
0.0969	0.1642	0.7389	0.9280	0.0641	0.0079
0.1391	0.2390	0.6219	0.9294	0.0660	0.0046
0.2099	0.2879	0.5022	0.9086	0.0868	0.0046
0.2636	0.3212	0.4152	0.9427	0.0519	0.0054
0.3231	0.3582	0.3187	0.9320	0.0614	0.0066

**Table 3:** Experimental (liquid + liquid) equilibrium data for the ternary system of water (1) + diethyl carbonate (2) + benzene (3) for mole fractions *x* at *T* = 298.15 K

Organic phase			Aqueous phase		
*x*_1_	*x*_2_	*x*_3_	*x*_1_	*x*_2_	*x*_3_
0.0425	0.1234	0.8341	0.9933	0.0048	0.0019
0.0336	0.2285	0.7379	0.9926	0.0052	0.0022
0.0373	0.3003	0.6624	0.9910	0.0066	0.0024
0.0412	0.3852	0.5736	0.9906	0.0066	0.0028
0.0423	0.4666	0.4911	0.9922	0.0054	0.0024
0.0405	0.5097	0.4498	0.9934	0.0039	0.0027
0.0383	0.5634	0.3983	0.9951	0.0029	0.0020

**Table 4:** Experimental (liquid + liquid) equilibrium data for the ternary system of water (1) + diethyl carbonate (2) + cyclohexane (3) for mole fractions *x* at *T* = 298.15 K

Organic phase			Aqueous phase		
*x*_1_	*x*_2_	*x*_3_	*x*_1_	*x*_2_	*x*_3_
0.0463	0.0948	0.8589	0.9710	0.0019	0.0271
0.0347	0.1770	0.7883	0.9765	0.0014	0.0221
0.0528	0.2592	0.6880	0.9803	0.0012	0.0185
0.0587	0.3198	0.6215	0.9879	0.0013	0.0108
0.0472	0.3880	0.5648	0.9817	0.0014	0.0169
0.0498	0.4084	0.5418	0.9801	0.0015	0.0184
0.0440	0.4462	0.5098	0.9764	0.0018	0.0218
0.0475	0.4850	0.4675	0.9763	0.0016	0.0221
0.0502	0.5664	0.3834	0.9796	0.0020	0.0184
0.0445	0.6220	0.3335	0.9795	0.0019	0.0186

**Table 5:** Experimental (liquid + liquid) equilibrium data for the quaternary system of water (1) + propan-1-ol (2) + diethyl carbonate (3) + benzene (4) for mole fractions *x* at *T* = 298.15 K

Aqueous phase			Organic phase		
*x*_1_	*x*_2_	*x*_3_	*x*_1_	*x*_2_	*x*_3_
{*x*_1_water + *x*_2_propan-1-ol + *x*_3_diethyl carbonate + (1 – *x*_1_ – *x*_2_ – *x*_3_)benzene}^a^					
*x*_3_^′^ = 0.20^b^					
0.9642	0.0358	0.0000	0.0320	0.0913	0.2290
0.9523	0.0477	0.0000	0.0823	0.2097	0.1960
0.9518	0.0482	0.0000	0.0732	0.2653	0.1721
0.9387	0.0613	0.0000	0.1071	0.3293	0.1630
0.9222	0.0778	0.0000	0.1211	0.3706	0.1429
0.9320	0.0680	0.0000	0.1385	0.4185	0.1320
0.9235	0.0765	0.0000	0.1689	0.4432	0.1164
0.9290	0.0710	0.0000	0.1933	0.4734	0.1064
0.9207	0.0793	0.0000	0.2499	0.4535	0.0817
0.9244	0.0756	0.0000	0.2883	0.4394	0.0706
0.9243	0.0757	0.0000	0.3271	0.4334	0.0541
*x*_3_^′^ = 0.40^b^					
0.9306	0.0694	0.0000	0.0359	0.1274	0.4270
0.9204	0.0796	0.0000	0.0674	0.2129	0.3407
0.9030	0.0970	0.0000	0.0728	0.2841	0.3150
0.9278	0.0722	0.0000	0.1049	0.3029	0.3050
0.8995	0.1005	0.0000	0.1446	0.3830	0.2328
0.9009	0.0991	0.0000	0.1611	0.4089	0.2209
0.8756	0.1244	0.0000	0.2080	0.4454	0.1701
0.9105	0.0895	0.0000	0.2753	0.4571	0.1216
*x*_3_^′^ = 0.60^b^					
0.9175	0.0801	0.0023	0.0598	0.0882	0.5652
0.9073	0.0907	0.0020	0.1036	0.1666	0.4888
0.9112	0.0872	0.0016	0.1027	0.2279	0.4832
0.8924	0.1059	0.0017	0.1548	0.2870	0.3883
0.9075	0.0909	0.0015	0.2089	0.3331	0.3070
0.9022	0.0959	0.0019	0.2524	0.3784	0.2222
0.8736	0.1243	0.0022	0.3048	0.3875	0.1723
0.8731	0.1240	0.0029	0.3332	0.4033	0.1521
0.8881	0.1095	0.0024	0.4505	0.3714	0.0968
*x*_3_^′^ = 0.80^b^					
0.8765	0.1196	0.0039	0.4619	0.3693	0.1195
0.8771	0.1192	0.0037	0.5200	0.3570	0.0837
0.8697	0.1268	0.0035	0.5148	0.3504	0.0910
0.8823	0.1140	0.0037	0.5683	0.3271	0.0656
0.8737	0.1223	0.0040	0.5794	0.3206	0.0661
0.8941	0.1002	0.0057	0.1835	0.2744	0.4418
0.8948	0.1019	0.0033	0.2520	0.3396	0.3213

^a^Obtained by mixing pure water and propan-1-ol with the binary mixtures of {*x*_3_^′^ DEC + (1 – *x*_3_^′^) benzene}

^b^Mole fraction ratio of DEC and benzene in the binary mixtures

**Table 6:** Experimental (liquid + liquid) equilibrium data for the quaternary system of water (1) + propan-1-ol (2) + diethyl carbonate (3) + cyclohexane (4) for mole fractions *x* at *T* = 298.15 K

Aqueous phase			Organic phase		
*x*_1_	*x*_2_	*x*_3_	*x*_1_	*x*_2_	*x*_3_
{*x*_1_water + *x*_2_propan-1-ol + *x*_3_ diethyl carbonate + (1 – *x*_1_ – *x*_2_ – *x*_3_) cyclohexane}^a^					
*x*_3_^′^ = 0.20^b^					
0.9471	0.0486	0.0043	0.0152	0.0137	0.1937
0.9427	0.0531	0.0042	0.0174	0.0469	0.1758
0.9268	0.0691	0.0041	0.0286	0.0901	0.1642
0.9251	0.0729	0.0021	0.0387	0.1523	0.1300
0.9204	0.0776	0.0019	0.0589	0.2094	0.1271
0.9151	0.0828	0.0021	0.1097	0.2440	0.1069
0.9165	0.0818	0.0017	0.1741	0.2557	0.0889
0.9177	0.0807	0.0016	0.1984	0.2499	0.0795
*x*_3_^′^ = 0.40^b^					
0.9525	0.0430	0.0045	0.0283	0.0361	0.3716
0.9418	0.0542	0.0040	0.0390	0.0636	0.3458
0.9338	0.0633	0.0029	0.0347	0.1271	0.3111
0.9411	0.0567	0.0021	0.0810	0.1874	0.2750
0.9290	0.0691	0.0019	0.1437	0.2205	0.2246
0.9284	0.0698	0.0019	0.1771	0.2444	0.1925
0.9259	0.0722	0.0019	0.2178	0.2778	0.1669
0.9257	0.0722	0.0021	0.2484	0.2827	0.1422
*x*_3_^′^ = 0.60^b^					
0.9602	0.0374	0.0024	0.0472	0.0455	0.5430
0.9463	0.0517	0.0019	0.0593	0.0820	0.5452
0.9381	0.0595	0.0024	0.0612	0.1471	0.4862
0.9359	0.0616	0.0025	0.1444	0.1789	0.3642
0.9369	0.0612	0.0019	0.1974	0.2402	0.3246
0.9401	0.0579	0.0020	0.2284	0.2547	0.2710
0.9311	0.0671	0.0018	0.2512	0.2717	0.2316
0.9280	0.0692	0.0028	0.2874	0.2836	0.1957
*x*_3_^′^ = 0.80^b^					
0.9643	0.0325	0.0032	0.0886	0.0491	0.7099
0.9552	0.0419	0.0029	0.1190	0.0957	0.6311
0.9458	0.0512	0.0030	0.1538	0.1502	0.5490
0.9399	0.0572	0.0029	0.2087	0.1965	0.4566
0.9364	0.0562	0.0074	0.2413	0.2405	0.3934
0.9363	0.0585	0.0051	0.2826	0.2546	0.3359
0.9308	0.0643	0.0049	0.3234	0.2711	0.2878
0.9213	0.0750	0.0037	0.3673	0.2787	0.2441

^a^Obtained by mixing pure water and propan-1-ol with the binary mixtures of {*x*_3_^′^ DEC + (1 – *x*_3_^′^) cyclohexane}

^b^Mole fraction ratio of DEC and cyclohexane in the binary mixtures

**Table 7:** Experimental (liquid + liquid) equilibrium data for the quaternary system of water (1) + propan-1-ol (2) + diethyl carbonate (3) + heptane (4) for mole fractions *x* at *T* = 298.15 K

Aqueous phase			Organic phase		
*x*_1_	*x*_2_	*x*_3_	*x*_1_	*x*_2_	*x*_3_
{*x*_1_water + *x*_2_propan-1-ol + *x*_3_diethyl carbonate + (1 – *x*_1_ – *x*_2_ – *x*_3_) heptane}^a^					
*x*_3_^′^ = 0.20^b^					
0.9514	0.0460	0.0000	0.0308	0.0950	0.2079
0.9286	0.0714	0.0000	0.0411	0.2587	0.1657
0.8436	0.1564	0.0000	0.0602	0.3829	0.1323
0.9098	0.0902	0.0000	0.0705	0.4148	0.1247
0.8961	0.1039	0.0000	0.1226	0.4816	0.0854
0.8383	0.1617	0.0000	0.1686	0.4842	0.1047
0.8160	0.1840	0.0000	0.2093	0.5007	0.0729
0.8337	0.1663	0.0000	0.2195	0.5072	0.0642
0.8651	0.1349	0.0000	0.3001	0.5006	0.0439
*x*_3_^′^ = 0.40^b^					
0.9181	0.0724	0.0046	0.0312	0.1102	0.5550
0.9474	0.0440	0.0040	0.0387	0.2009	0.4438
0.9037	0.0879	0.0021	0.0526	0.2957	0.3276
0.9019	0.0901	0.0020	0.0950	0.3869	0.2509
0.9042	0.0892	0.0019	0.1425	0.4641	0.1865
0.8989	0.0942	0.0020	0.2122	0.4779	0.1498
0.8933	0.0993	0.0030	0.2326	0.5001	0.1183
0.8834	0.1095	0.0026	0.2766	0.5009	0.0983
*x*_3_^′^ = 0.60^b^					
0.9549	0.0330	0.0054	0.0454	0.0590	0.5137
0.9436	0.0465	0.0039	0.0451	0.1232	0.4845
0.9357	0.0547	0.0040	0.0676	0.1969	0.4218
0.9256	0.0646	0.0038	0.1079	0.2550	0.3637
0.9262	0.0623	0.0062	0.1586	0.3220	0.2947
0.9188	0.0683	0.0054	0.2212	0.3412	0.2482
0.9140	0.0742	0.0051	0.2579	0.3603	0.2126
0.9166	0.0740	0.0050	0.2859	0.3793	0.1837
*x*_3_^′^ = 0.80^b^					
0.9524	0.0374	0.0040	0.0612	0.0868	0.6616
0.9449	0.0457	0.0040	0.0763	0.1368	0.6154
0.9363	0.0533	0.0037	0.1336	0.2068	0.5077
0.9341	0.0577	0.0034	0.1761	0.2599	0.4263
0.9276	0.0630	0.0030	0.2423	0.3041	0.3459
0.9244	0.0652	0.0037	0.2789	0.3257	0.2948
0.9210	0.0679	0.0041	0.3033	0.3476	0.2612
0.8899	0.0970	0.0052	0.3398	0.3524	0.2267

^a^Obtained by mixing pure water and propan-1-ol with the binary mixtures of {*x*_3_^′^ DEC + (1 – *x*_3_^′^) heptane}

^b^Mole fraction ratio of DEC and heptane in the binary mixtures

The modified UNIQUAC and extended UNIQUAC models were employed to correlate the experimental LLE data. For most multicomponent systems, especially for type 1 systems having a plait point, the original UNIQUAC model with only two binary parameters did not always give accurate results. So, in order to accurately correlate ternary and quaternary LLE, it is necessary to use ternary and quaternary parameters in addition to the binary ones. The ternary and quaternary parameters were determined from the experimental LLE data using a simplex method [18] by minimizing the function *F*:

1 $$ F = 100 \cdot \left\{ {\sum\limits_{k} {\sum\limits_{i} {\sum\limits_{j} {\left( {x_{ijk}^{ \exp } - x_{ijk}^{\text{cal}} } \right)^{2} /2ni} } } \}^{0.5} } \right\} $$

where *x* denotes the mole fraction in the liquid phase, *i* = 1 to 3 for a ternary system or *i* = 1 to 4 for a quaternary system, and *j* = 1, 2 (phases), *k* = 1, 2, …, *n*, where *n* stands for the number of tie lines as shown in Tables 10 and 11. Here exp denotes experimental values, and cal denotes calculated values of the models.

Table 8 shows the molecular structural volume and area parameters, where *r* and *q* are taken from the literature [5, 19]. The interaction correction factors *q*′, for nonassociating components such as DEC, benzene, cyclohexane and heptanes, were set to *q*′ = *q* ^0.75^ in the modified UNIQUAC model and *q*′ = *q* ^0.20^ in the extended UNIQUAC model, while those for associating components such as water and propan-1-ol were taken from the literature [7, 8].

**Table 8:** Structural parameters for pure components

Component	*r* ^a^	*q* ^a^	*q*′^b^	*q*′^c^
Benzene	3.19	2.40	q^0.75^	q^0.20^
Cyclohexane	3.97	3.01	q^0.75^	q^0.20^
DEC	4.41	3.90	*q* ^0.75^	*q* ^0.20^
Heptane	5.17	4.40	q^0.75^	q^0.20^
Propan-1-ol	2.78	2.51	1.32	0.89
Water	0.92	1.40	1.28	0.96

^a^From references [5, 19]

^b^Modified UNIQUAC model

^c^Extended UNIQUAC model

Table 9 lists the binary parameters *a*_*ij*_ of the modified UNIQUAC and extended UNIQUAC models for the constituent binary mixtures, along with the standard deviations between experimental and calculated values: *δ*(*p*) for pressure, *δ*(*T*) for temperature, *δ*(*x*) for liquid phase mole fraction, and *δ*(*y*) for vapor phase mole fraction. Good agreement was obtained between experimental results and those calculated by both models.

**Table 9:** Calculated results from binary phase equilibrium data reduction

System(1 + 2)	*a*_12_/K	*a*_21_/K	*δ*(*P*)^c^/kPa	*δ*(*T*)^c^/K	10^3^ *δ*(*x*)^c^	10^3^ *δ*(*y*)^c^	lit.
Propan-1-ol + water	159.06^a^ 138.20^b^	262.46 244.51	1.49 1.52	0.11 0.11	2.80 2.90	6.70 6.80	[9]
Propan-1-ol + DEC	75.71^a^ 97.62^b^	175.10 176.76	1.90 1.90	0.12 0.12	2.50 2.50	3.00 3.00	[10]
Benzene + propan-1-ol	606.64^a^ 586.62^b^	92.98 91.70	1.45 1.46	0.05 0.05	0.60 0.60	5.40 5.40	[11]
DEC + benzene	–193.28^a^ –160.28^b^	191.39 157.75	1.76 1.90	0.01 0.01	0.60 0.70	8.60 9.40	[12]
Cyclohexane + propan-1-ol	1018.29^a^ 965.40^b^	110.86 122.69	0.87 0.92	0.03 0.03	0.30 0.40	4.30 4.50	[11]
Cyclohexane + DEC	–52.53^a^ –47.09^b^	211.09 230.09	2.55 2.54	0.14 0.14	2.90 2.80	8.10 8.10	[13]
Heptane + DEC	144.20^a^ 181.84^b^	31.22 69.08	1.30 1.30	0.07 0.07	0.80 0.80	8.00 7.90	[13]
Heptane + propan-1-ol	757.15^a^ 738.64^b^	117.88 164.47	4.30 4.32	0.19 0.19	4.40 4.40	17.00 16.90	[14]
Water + cyclohexane	1157.80^a^ 1315.60^b^	2429.90 1942.50					[15]
Water + DEC	248.21^a^ 273.66^b^	1177.60 961.41					[16]
Water + heptane	1022.10^a^ 1839.60^b^	1884.20 2135.50					[17]
Water + benzene	762.26^a^ 750.12^b^	1663.80 1365.30					[17]

^a^Modified UNIQUAC model

^b^Extended UNIQUAC model

^c^Root-mean-square deviation

**Fig. 3:**
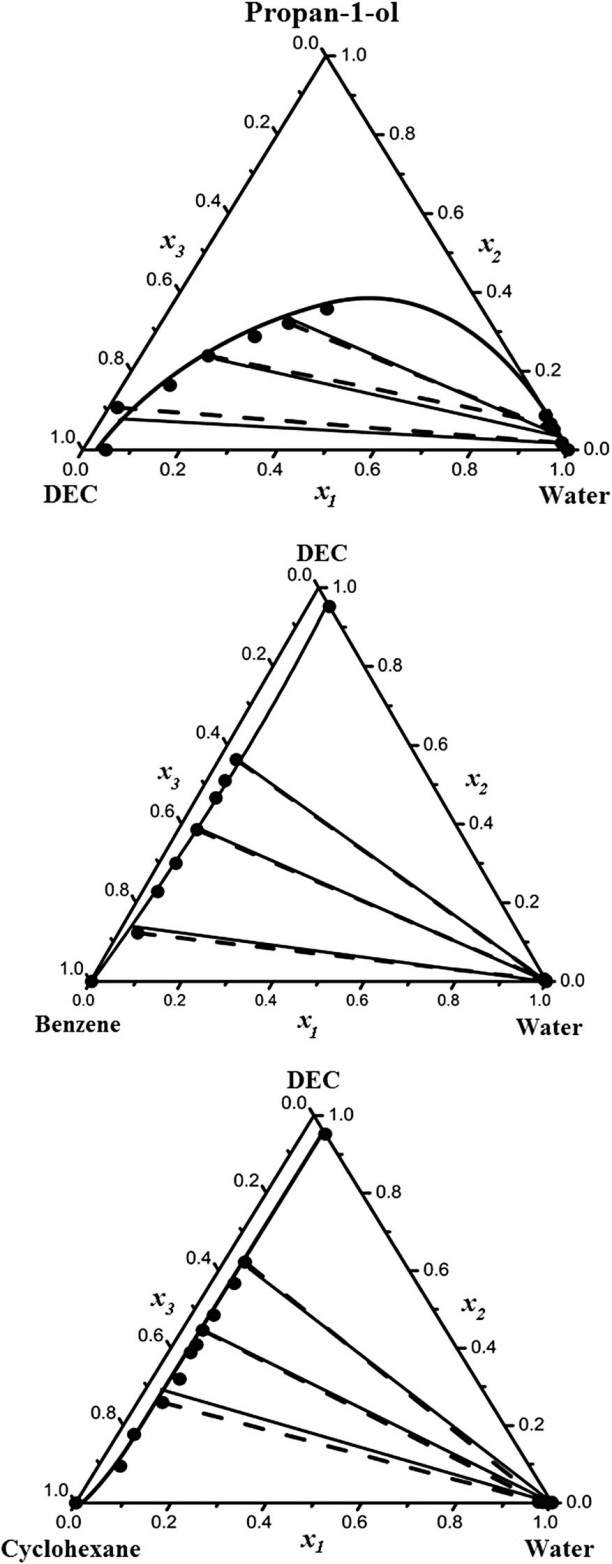
Experimental and calculated LLE of ternary systems (water + propan-1-ol + DEC), (water + propan-1-ol + benzene) and (water + propan-1-ol + cyclohexane) at *T* = 298.15 K. *filled circle*, Experimental tie-line data; *lines*, predicted results by the modified UNIQUAC model using binary parameters taken from Table 9; *dotted lines*, correlated results by the modified UNIQUAC model using binary and ternary parameters taken from Tables 9 and 10

Table 10 presents the ternary mixture parameters, *τ*_231_, *τ*_132_ and *τ*_123_, together with the root-mean-square deviation (rmsd) values between the experimental and calculated tie lines for the ternary LLE. The comparison is shown on the phase diagram in Fig. 3 by means of the experimental and calculated tie lines for the three ternary systems (water + propan-1-ol + DEC), (water + propan-1-ol + benzene) and (water + propan-1-ol + cyclohexane). This figure indirectly illustrates the accuracy of the experimental LLE data. As shown in this figure, excellent correlation is seen by the extended and modified UNIQUAC models. For the three ternary systems investigated in this work, the average rmsd values of correlated results are 0.88 and 1.03 % for the modified and extended UNIQUAC models, respectively. Good agreement between the experimental and correlated tie line data of the two models is indicated by the low rmsd values.

**Table 10:** Calculated results of ternary liquid–liquid equilibria

System (1 + 2 + 3)	*n* ^a^	*τ*_231_	*τ*_132_	*τ*_123_	rms^d,e^	rms^d,f^	lit.
Water + propan-1-ol + DEC	7	0.0342^b^ –0.0063^c^	0.6215 –0.3134	1.5495 0.8005	3.61 1.20	0.76 1.07	This work
Water + DEC + benzene	7	0.1465^b^ 0.0914^c^	0.1834 0.1165	–0.1575 –0.0483	1.31 1.03	0.60 0.67	This work
Water + DEC + cyclohexane	10	0.1018^b^ 0.1554^c^	4.3906 0.1448	–1.2452 –0.0094	2.00 1.82	1.27 1.34	This work
water + propan-1-ol + benzene	12	0.0011^b^ 5.0259^c^	0.0009 –4.1570	0.9061 0.8381	2.79 3.60	2.12 2.57	[20]
Water + propan-1-ol + cyclohexane	7	0.0488^b^ 0.0059^*c*^	–1.3082 0.6180	2.8434 0.2023	2.54 3.05	1.41 1.79	[15]
Water + propan-1-ol + heptane	11	0.0217^b^ 0.1289^c^	–1.5764 –1.1171	0.1941 0.2031	18.61 16.65	2.90 2.42	[20]
Water + DEC + heptane	13	–0.0333^b^ –0.0509^c^	0.1576 0.1864	–0.0175 –0.8319	0.85 1.31	0.26 0.65	[16]

^a^Number of tie lines

^b^Modified UNIQUAC model

^c^Extended UNIQUAC model

^d^Root-mean-square deviation (mol-%)

^e^Predicted results using binary parameters taken from the Table 9

^f^Correlated results using binary and ternary parameters

Table 11 summarizes the quaternary parameters, *τ*_2341_, *τ*_1342_, *τ*_1243_ and *τ*_1234_, together with the correlated results obtained by fitting the modified and extended UNIQUAC models with binary, ternary, and quaternary parameters to the experimental quaternary LLE data, together with the predicted results by the models with only the binary and ternary parameters listed in Tables 9 and 10. For the three investigated quaternary systems, the average rmsd values of correlated results are 2.68 and 2.96 % for the extended and modified UNIQUAC models, respectively. It can be seen that the correlated results obtained from both models are better than the predicted ones in representing the quaternary LLE measured in this work. This is due to adding the quaternary parameters in the correlation.

**Table 11:** Calculated results of quaternary liquid–liquid equilibria

system (1 + 2 + 3 + 4)	*n* ^a^	*τ*_2341_	*τ*_1342_	*τ*_1243_	*τ*_1234_	Rms^d,e^	Rms^d,f^
Water + propan-1-ol + DEC + benzene	35	1.7213^b^ 0.1941^c^	–25.3641 –20.5046	–4.9044 –2.9720	–1.3272 0.0844	4.76 5.42	2.59 2.86
Water + propan-1-ol + DEC + cyclohexane	32	1.3683^b^ –4.9950^c^	–4.7834 19.8699	–1.2105 –15.6978	–1.0180 2.5136	4.66 4.22	2.65 1.21
Water + propan-1-ol + DEC + heptane	31	0.1506^b^ 0.0319^c^	–0.6409 –0.5804	–1.3521 –2.6306	0.4257 0.8480	4.24 7.34	2.79 2.80

^a^Number of tie lines

^b^Modified UNIQUAC model

^c^Extended UNIQUAC model

^d^Root-mean-square deviation (mol-%)

^e^Predicted results using binary and ternary parameters taken from the Tables 9 and 10

^f^Correlated results using binary, ternary and quaternary parameters

**Fig. 4:**
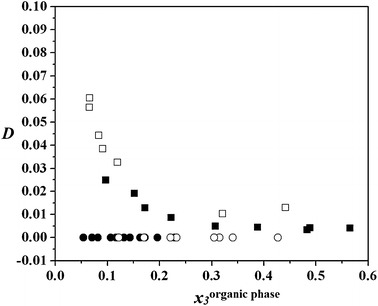
Distribution coefficient, *D*, of DEC in the quaternary system of water (1) + propan-1-ol (2) + DEC (3) + benzene (4) as a function of mole fraction of DEC in the organic-rich phase, *x*_3_: *filled circle*, *white circle*, *filled square*, *white square*, *x*_3_^′^ = 0.2, 0.4, 0.6, and 0.8, respectively

**Fig. 5:**
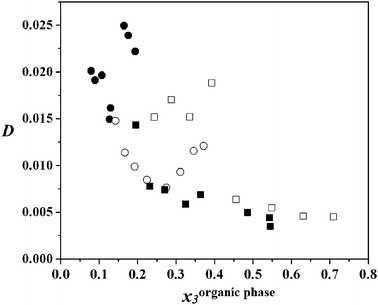
Distribution coefficient, *D*, of DEC in the quaternary system of water (1) + propan-1-ol (2) + DEC (3) + cyclohexane (4), as a function of mole fraction of DEC in the organic-rich phase, *x*_3_: *filled circle*, *white circle*, *filled square*, *white square*, *x*_3_^′^ = 0.2, 0.4, 0.6, and 0.8, respectively

**Fig. 6:**
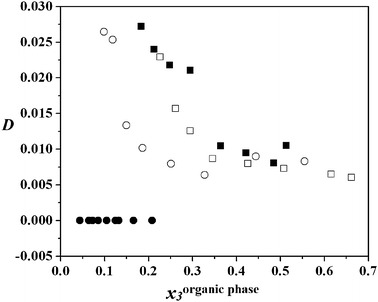
Distribution coefficient, *D*, of DEC in the quaternary system of water (1) + propan-1-ol (2) + DEC (3) + heptane (4), as a function of mole fraction of DEC in the organic-rich phase, *x*_3_: *filled circle*, *white circle*, *filled square*, *white square*, *x*_3_^′^ = 0.2, 0.4, 0.6, and 0.8, respectively

The equilibrium distribution coefficient of DEC, calculated from the experimental LLE data, is defined as: the ratio of the concentration of DEC in the aqueous phase to the concentration in the organic phase:

2 $$ D = x_{ 3}^{\rm{aqueous\;phase}} /x_{ 3}^{\rm{organic\;phase}} $$

where *D* is equilibrium distribution coefficient of DEC and *x*_3_is the mole fraction of DEC. Figures 4, 5 and 6 show the equilibrium distribution coefficient of DEC for the quaternary systems (water + propan-1-ol + DEC + benzene), (water + propan-1-ol + DEC + cyclohexane) and (water + propan-1-ol + DEC + heptane), at four different distribution ratio of *x*_3_ = 0.2, 0.4, 0.6, and 0.8. For the three measured quaternary systems, the equilibrium distribution coefficients of DEC show low values as shown in Figs. 4, 5 and 6. It can be concluded that adding DEC does not result in an evident increase of solubility of DEC in the aqueous phase. Since DEC has two ethyl groups, while alkyl is a hydrophobic group, DEC is more soluble in the organic phase.

## Conclusions

Under atmospheric pressure, the experimental LLE data for the ternary systems (water + propan-1-ol + DEC), (water + propan-1-ol + benzene) and (water + propan-1-ol + cyclohexane), and quaternary systems of (water + propan-1-ol + DEC + benzene), (water + propan-1-ol + DEC + cyclohexane) and (water + propan-1-ol + DEC + heptane) were obtained at *T* = 298.15 K. The experimental LLE results were successfully correlated using the extended and modified UNIQUAC models. The correlated results obtained by using the quaternary parameters as well as the binary and ternary parameters, with a rmsd value of less than 3 mol- %, showed good agreement with the experimental LLE results.
